# Human rights-based accountability for sexual and reproductive health and rights in humanitarian settings: Findings from a pilot study in northern Uganda

**DOI:** 10.1371/journal.pgph.0000836

**Published:** 2022-08-22

**Authors:** Grady Arnott, Charles Otema, Godfrey Obalim, Beatrice Odallo, Teddy Nakubulwa, Sam B. T. Okello

**Affiliations:** 1 Center for Reproductive Rights, Global Legal Program, New York, New York, United States of America; 2 CARE International in Uganda, Health Equity and Rights Team, Bugolobi, Kampala, Uganda; 3 Center for Reproductive Rights, Global Legal Program, Nairobi, Kenya; 4 CARE International in Uganda, Gender Justice Program, Bugolobi, Kampala, Uganda; Jhpiego, UNITED STATES

## Abstract

Ensuring accountability for the realization of sexual and reproductive health and rights is a human rights obligation and central tenet of strategies to improve health systems and outcomes in humanitarian settings. This pilot study explored the feasibility and acceptability of deploying human rights strategies, specifically through a participatory community-led complaints mechanism, to hold humanitarian health systems to account for the sexual and reproductive health and rights of refugee and host community women and girls in northern Uganda. Over a fifteen-month period we conducted a multi-methods exploratory study with refugee and host community rights-holders and duty-bearers using longitudinal in-depth interviews, focus groups, and secondary data document review. Deductive and inductive coding techniques were used to analyze data iteratively for content and themes. 107 sexual and reproductive health and rights related complaints and feedback were collected through the community complaints mechanism. Complaints concerned experiences of disrespect and abuse by health care workers; lack of adolescent access to sexual and reproductive health services and information; sexual and gender-based violence; and lack of access to acceptable and quality health goods and services. Participants reported an increased understanding and claiming of human rights through the intervention, acceptability of rights-based accountability strategies among humanitarian health system actors, and improved access to remedies when sexual and reproductive health rights are not respected. Findings demonstrate integrating rights-based social accountability mechanisms at the level of humanitarian response as a promising approach for strengthening and holding humanitarian health systems accountable for the sexual and reproductive health and rights of women and girls affected by humanitarian situations.

## Introduction

Increasing protraction of conflict, human rights violations, and climate-related disasters continue to drive global forced displacement trends [1]. An estimated 35 million women of reproductive age and 29 million adolescents and young people require humanitarian assistance, and both need and have equal rights to essential and lifesaving sexual and reproductive health (SRH) services that are often limited in humanitarian settings [2–4]. Research in these contexts indicates more stigmatized SRH services such as access to comprehensive abortion care and adolescent sexual and reproductive health and rights (SRHR) are even more limited [5, 6]. Moreover, many pre-existing forms of intersectional discrimination and inequities that are compounded by humanitarian crises have been deepened by the COVID-19 pandemic, including harmful social and gender norms and systematic risk faced by affected women and girls [7–9].

Ensuring accountability for the realization of SRHR, including in humanitarian settings, is a human rights obligation [4, 5]; and a central tenet of strategies to reduce maternal mortality and morbidity, improve health systems and outcomes, and deliver on health and human rights commitments [10–13]. Promoting accountability for SRHR is achieved through a range of strategies, including legal and policy mechanisms; performance measurement and impact standards; and social or community accountability processes wherein duty-bearers are held to account through collective efforts to meet existing obligations to provide goods and services [14–17]. Existing evidence demonstrates how context-specific accountability strategies are needed to account for the complexity of power dynamics that are relevant in all settings, but acutely so in humanitarian settings and in the context of displacement [18].

Human rights-based accountability requires multiple forms and approaches to ensure that accountability is not an afterthought after human rights violations occur. The *UN Technical Guidance on the application of a human-rights based approach to implementation of policies and programmes to reduce preventable maternal morbidity and mortality* conceptualizes a “circle of accountability” to promote accountability through diverse and participatory forms of planning, implementation, and monitoring within the humanitarian program cycle [19]. Moving beyond a checklist process, rights-based accountability seeks to transform health systems and sustain change through acknowledging contextual and political factors, power dynamics, processes of contestation, and need for meaningful participation to realize SRHR [18, 20, 21]. However, demanding accountability and claiming rights through formal channels may be especially difficult for women and girls affected by humanitarian crises who may face a myriad of risk and reprisal for openly discussing SRHR and participating in decision-making processes in contravention of harmful social and gender norms and stereotypes [16, 22, 23]. A level of risk is also assumed given reliance on the host state and the humanitarian system for essential and lifesaving services, as is the case with refugee women and girls displaced from South Sudan and Ugandan nationals residing in post-conflict northern Uganda.

Over five decades of armed conflict and political instability has led to severe and protracted humanitarian crises in South Sudan. Despite the September 2018 signing of the Revitalized Peace Agreement on the Resolution of the Conflict in South Sudan (R-ARCSS) and a reduction in violence, the political system has not been reformed, health infrastructure and access to timely and quality SRH services is inadequate for internally displaced persons (IDPs), and sexual violence remains widespread and pervasive [24]. As one of the leading refugee hosting countries in the world, Uganda hosts 1.5 million refugees of which 925,499 are displaced from South Sudan [25]. The Comprehensive Refugee Response Framework in Uganda (CRRF) and Refugee Coordination Model between the government and the United Nations High Commissioner for Refugees (UNHCR) sets out access to health services for refugees; the integrated model is used to deliver services through the district health system to both refugees and host populations with the support of humanitarian organizations [26–28].

Several studies have documented the service and systems-level barriers that affect refugee and host women and girls’ access to SRH services and information in Uganda, including the influence of social and gender norms on unmet need for contraceptives; poor referral systems for delivery of quality emergency obstetric and neonatal care (EmONC); restrictive abortion laws and policies and limited provision of lawful abortion in the settlements; and lack of confidential and acceptable approaches to SRH service delivery to adolescents [29–35].

Research on social accountability interventions in Uganda demonstrates how social accountability approaches, such as community scorecards, community dialogues, citizen monitoring, and budget audits, can improve the quality of SRH services, increase service utilization, build trust between community and health systems actors and embed legitimized social accountability processes through institutionalization within existing systems, processes, or policies [36–40]. Yet, few interventions implement strategies that expressly adopt a human rights-based approach; that is are designed to and document how human rights standards can be leveraged within the social accountability process. Social accountability initiatives that do engage with human rights law and policy to effect duty-bearer response often concentrate on rights and entitlements under national legal frameworks, rather than the international human rights system. SRHR is enshrined in a constellation of rights in human rights treaties and jurisprudence from UN treaty monitoring bodies provides robust standards on access to SRHR services and information, including in humanitarian settings where the breakdown of national legal and health infrastructure continues to impede access to quality services. Application of international standards within the social accountability process and intervention design may be a key strategy to ensure SRHR is considered comprehensively and that marginalized populations and stigmatized issues are not excluded from the social accountability process.

This paper responds to calls for more research on approaches to strengthen accountability for SRHR and specifically its application at the local level in humanitarian settings. Furthermore, it seeks to demonstrate how leveraging and integrating international human rights standards more expressly into social accountability processes can bring value and achieve key SRHR outcomes in the context of integrated humanitarian health systems. Drawing from a pilot study implemented in Pagirinya refugee settlement in northern Uganda, this paper sets out the feasibility and acceptability of deploying human rights strategies, specifically through a participatory community-led complaints mechanism, to hold humanitarian health systems to account for the SRHR of refugee and host community women and girls.

## Materials and methods

To address research and practice gaps we conducted a multi-methods exploratory study to assess the feasibility and acceptability of integrating rights-based social accountability mechanisms within refugee response programming. Between March 2020 –June 2021 the intervention was piloted in Pagirinya refugee settlement and surrounding host community located in Adjumani district, northern Uganda (S1 Fig). The study site was purposively selected based on the existing SRHR programming led by one of the study partner organizations and given that Adjumani hosts one of the largest populations of refugees in Uganda [25, 41]. The intervention was designed using a participatory approach with refugee and host women and girls, including consultative mapping of their access to the existing accountability ecosystem in the settlement and identification of leaders from women, adolescent, and disability communities for engagement in the intervention structures. Consultations with duty-bearers from district level government departments, humanitarian and district health systems, UN agencies, and national and international humanitarian implementing organizations were conducted to inform opportunities for coordination and uptake in the district. For the purposes of study design, data collection, and analysis, participants were defined and categorized as either *rights-holders* as persons with existing rights and entitlements, or *duty-bearers* as actors with obligations to respect, protect, and fulfill SRHR [42].

The purpose of the intervention was to embed a social accountability mechanism within humanitarian health systems and coordinating mechanisms to improve refugee and host community women and girls’ access to SRH services and ensure access to effective remedies when SRHR is not respected. The resulting model comprised an integrated three-part community-level complaints mechanism, including a Community Council to collect and review SRHR-related complaints and feedback; Ombudsperson to work with duty-bearers to respond to complaints using dialogues, direct advocacy, or referral processes; and Community Monitors to track implementation of effective remedies at the settlement level. S1 Table sets out a detailed summary of the intervention components, participant sample, recruitment strategy, mandate of each component, and key data sources generated through their activities. All three structures created by the intervention: the Council for SRHR, Ombudsperson, and Community Monitors were trained on human rights-based approaches to SRHR and accountability, with a focus on the application of human rights standards and principles in the context of SRH service delivery. Strategies for engaging with duty-bearers and ethical data monitoring were also part of an initial and refresher training midway through the intervention.

### Data collection

Over a fifteen-month period, longitudinal in-depth interviews (n = 50) and focus groups discussions (n = 14) were conducted. Rights-holders were sampled from health or social programming in the settlement and informal community women and adolescent peer-groups. Duty-bearers were sampled from ten district government departments based on the results of the accountability ecosystem mapping during the design phase and monthly engagement with the Ombudsperson. In-depth interviews (n = 6) and focus group discussions (n = 14) with rights-holders explored the respondents’ experiences with, opinions of, and perspectives on the availability, accessibility, acceptability, and quality of SRH services and information in the settlement; and satisfaction with the community complaints mechanism, if accessed. Interviews with duty-bearers (n = 44) focused on understanding their perspectives of their SRHR obligations, their position on institutionalizing core components of the rights-based accountability mechanism, and the factors that enable or impede them from discharging their duties to fulfill SRHR. Interviews and focus groups were conducted in participants’ language of choice (English, Madi, or Arabic) and lasted an average of 60 minutes. Interviewers and facilitators took detailed written notes during the research activity and audio recorded with participant consent.

Programmatic materials (n = 89) were collected for document review, including entries in the complaints’ logbook, public statements delivered by participating duty-bearers, and fieldnotes and memos documented by the intervention participants. Documents were collected and organized chronologically and routinely monitored for discussion during bi-weekly study team meetings.

### Data analysis

Audio recordings from in-depth open-ended interviews and focus groups were translated, as needed, by local research assistants, and transcribed verbatim into English. Secondary project documents were reviewed iteratively, categorized by phase of pilot implementation and classification (e.g., training resources, monthly activity reports, complaint logbook), and extracted through coding. *A priori* codes were developed based on the research question, core components of a human rights-based approach, and definitions of SRHR and social accountability. Inductive codes that emerged throughout analysis were added to the list of codes and organized into assigned categories in a code book, which was used to code all data iteratively and again at endline. Analysis was iterative and emerging codes and category groupings were reviewed as data was collected and validated during bi-weekly study team meetings. The bi-weekly meetings were also used to review new entries to the complaint logbook, ensure consistency across multiple data sources, and guide thematic analysis. During the research period, the study team held three workshops to draw connections between ideas and discuss adaptations to improve implementation and respond to feedback from the participants. NVivo 12 Pro was used for data management throughout the research period and to code the primary and secondary data.

### Ethics statement

Ethics approval for this research was provided by Mildmay Uganda Research Ethics Committee (#REC REF 0505–2021). The intervention was also approved by the Office of the Prime Minister in Uganda and Office of the Camp in Charge. Written consent was obtained for in-person interviews and focus groups, and verbal consent for virtual interviews. Personally identifying information has been masked or removed from the data presented in this paper.

## Results

### Participant sample and complaint characteristics

The intervention reached 4,479 refugee and host community women and girls and within this sample collected 107 SRHR-related complaints. 70.09% (n = 75) of complaints were established to be related to SRHR and within the scope of the community complaints mechanism and 29.91% (n = 32) of complaints were referred to other accountability processes at district, sub-county, or refugee settlement level. Of the SRHR complaints, 70.67% (n = 53) were resolved directly through the intervention structures and 29.33% (n = 22) were still pending at pilot closure. Tracking outcomes of complaints referred out of the community mechanism was possible for 31.25% (n = 10) of cases; however, 50% (n = 16) were still open at the end of piloting and 18.75% (n = 6) were lost-to-follow-up. Most SRHR-related complaints were characterized by disrespect or abuse by health care workers while accessing antenatal care (ANC) and delivery services (n = 21). The next most frequently reported complaints concerned lack of adolescent access to SRH services and information (n = 19) and cases of SGBV (n = 18). SGBV cases were referred to the settlement health center and subsequently local protection actors on request of the survivor. Complaints characterized by lack of access to culturally and gender acceptable contraceptive counselling and quality goods and services (n = 7), HIV/STI counselling and treatment (n = 2), accessible delivery services for persons with disabilities (n = 2), and need to resort to unsafe abortion (n = 1) were also documented. Structural barriers were reported, such as lack of transport and administrative errors on refugee attestation cards, preventing equitable access to the health facility (n = 9). The remaining complaints collected by the intervention structures (n = 28) concerned issues outside the scope of the SRHR-focused pilot, such as food ration shortages, nutrition, and child protection matters, which were referred to district-level government, United Nations High Commissioner for Refugees (UNHCR), or responsible implementing partner mechanisms.

### Participatory community accountability structures facilitate an understanding and claiming of human rights

The intervention established multiple fora for complaints to be channeled, such as one-on-one confidential consultations with SRHR Council members; monthly dialogues organized between the ombudsperson and duty-bearers; home visits in the refugee settlement by community-based monitors to educate and generate demand for SRH services and information; and interactive workshops and media within adolescent and disability community solidarity peer groups. Responses from rights-holders and duty-bearers during in-depth interviews expressed how creating environments for confidential and non-stigmatizing dialogue about SRHR was an important feature of the intervention and affected participation among women and girls. Refugee and host women and girls revealed that low uptake of existing complaint mechanisms in the settlement (e.g., physical feedback boxes and hotlines) was due to low levels of literacy, confidentiality barriers, and as stated by one focus-group participant *“we see no change and no response*.*”* Adolescents and girls in particular expressed fear of reprisal for using public feedback boxes or asking questions about SRHR in a public forum that they perceived as “*not open to us*.”

The intervention structures focused on building a shared understanding of human rights, through the sensitization sessions and peer dialogues, including using media and dramatic performance within adolescent peer-groups. This approach enabled the community mechanism to ground complaints in existing human rights and entitlements, while supporting an open-ended collection system of participant-described and attributed problems and barriers. As described by a refugee women’s representatives on the SRHR Council: *“when we give awareness to the community … people knew their rights so they could demand for services at the health facility and for those services to be of quality*.*”*

Health sector duty-bearers perceived the role of SRHR representatives and the ombudsperson as successful for two main reasons: the trust given to them by the community and their ability to complete the feedback loop at individual and community level as part of the accountability process. The outcome of complaints was communicated directly back to the complainant or solidarity group where the complaint originated through the ombudsperson, or member of the SRHR Council. As illustrated by a technical lead from a national refugee response organization, this is distinct from existing complaints processes where *“information may not even reach the health facility and the feedback did not even reach the community*. *But with this project at least the communication is able to reach and the feedback is taken back to the community*.*”*

Duty-bearers that participated in monthly meetings with the ombudsperson and district-level health coordination meetings explained how articulation of SRH service delivery complaints led health system actors to acknowledge a process of service users understanding and claiming their rights; and thus, were influenced to take timely steps towards implementing changes. As described by a health sector senior clinical manager:

*“In the beginning we would get feedback about the quality of the health care services and particularly how mothers are tended to in the maternity unit*. *Now those would not come so clearly*, *and the community were not so empowered to bring them out… but during this project the community were able to bring out the utmost challenges they were facing in the facility when they go to access the SRH services*. *And the clarity in which they brought out those challenges enabled us to address those challenges more strongly and even in a faster way… so the greatest strength to me is the empowerment of the community to demand for the right to services and quality services that they are entitled to*. *And also empowerment of the service providers to appreciate that the community understands their rights and therefore the service delivery should be aligned with their expectations and based on the standards of quality that they have been empowered to demand*.*”*

District level government duty-bearers consistently expressed how the project “*brought us closer to the community*” and shaped their motivation for engagement in the response and remedy phase of the mechanism after seeing changes in community understanding and demands for SRHR. As expressed by an officer in the district-level government:

“*In the community we see that there is sensitization of their rights to have access to the services*. *When the issues are coming from the community then the duty-bearers*, *such as me*, *need to act*, *and the duty bearers from different sectors need to see that the issues come from the community*.*”*

An implementer from a national NGO attributed the intervention structures as the reason why sub-county and district leadership were becoming more involved in SRHR. The implementer recalled a change in witnessing duty-bearers *“going to the local health centers to talk about sexual and reproductive health and rights*. *That was not happening before*. *These local leaders are now being engaged to make them accountable*.*”* Another humanitarian program staff described how this change was the result of building the capacity of duty-bearers throughout the intervention to understand and meet their SRHR obligations, “*now the district leadership understand that they are actually accountable for SRH*. *That they have to ensure that their people can access sexual health and rights services*.*”*

Several humanitarian implementers perceived the participation of refugee and host women and girls directly in the operation of the accountability mechanism, and as a result other institutionalized decision-making processes in the settlement, as one of the most significant changes resulting from the intervention. Key informants from health service delivery organizations reflected that community structures, for example Village Health Teams (VHT) or Community-Based Facilitators (CBFs) are mainly mobilized to create awareness about and generate demand for SRH services and information, rather than as part of identifying gaps and monitoring service access and quality. As described by an adolescent SRHR program specialist working with youth populations:

*“This project had continuous engagement*. *We don’t use our groups formally*, *like this project has done*. *This was first of its kind*. *For us our projects are aimed at building capacity of volunteers to pass on information to women so that they can be in a position to access those services*, *but not identifying the gaps or the problems that hinder girls and women to reach the health center*.*”*

### Improving the availability, accessibility, acceptability, and quality of SRH services as an effective remedy when rights are not respected

Monthly dialogues between the ombudsperson and district-level government, UN agencies, and health system actors facilitated routine dialogue regarding complaints and exploration of effective redress measures. The meetings also generated a transparent public record of the anonymized complaints and identified responsible duty-bearers along with proposed strategies for redress and follow-up. The meeting minutes were revisited in subsequent monthly meetings, allowing the ombudsperson to follow up through the intervention structures and the community about actions taken. Changes resulting from the ombudsperson’s engagement included district-level revision of access to essential medicines policies to allow all incoming refugees to access antiretroviral treatment (ART) at the health center closest to their settlement. This change reduced transportation barriers and movement restrictions, which were compounded by COVID-19 pandemic measures that were in place throughout the intervention. The monthly meetings also led to renewed commitments from the district-level government to increase the frequency of oversight and monitoring visits to health facilities to assess the quality of services and hear from community women leaders and monitors. Aligning monthly meetings with institutionalized budget processes led by the Health Management Unit (HMU) and health center management, the ombudsperson held duty-bearers accountable to identify or create new budget lines for ensuring accessible and quality SRH services for persons with disabilities. Pregnant persons with disabilities delivering at the health center reported physical barriers in transferring to the delivery beds resulting in some deliveries occurring on the maternity ward floor. A budget line was identified to contract a local carpenter to design and install a supportive timber lift on the delivery beds. A Council member from the disability community expressed that the accessibility improvements marked a recognition of the rights of persons with disabilities:

“*Our voices are now being heard*. *If we go to the health facility to talk about ourselves*, *you find respect is given to us*. *Before people looked at us to be nothing*, *we were not being respected… and now I am able to talk about this [the concerns of the disability community] in public*.*”*

The accountability mechanism also established channels for direct advocacy between Council representatives and health center management actors. Inclusion of a health care worker on the Council for SRHR, who was nominated by peers and health center management, was important for opening dialogue between the community and health workers. This ensured health care workers were involved in the design of the intervention and prevented resistance given the representative could reinforce and clarify the purpose of the project in line with service delivery goals. As the intervention progressed, trust and collaboration between management-level decision-makers within the primary health system and Council members strengthened. As explained by an implementing partner leading family planning programming in the refugee health centers:

*“You find these women counsellors they are now free to come and have interactions with the in-charge of the facility where their community comes to get services*. *And there they can monitor… and when they find something is going astray they can work to immediately change it with the in-charge of the facilities*.*”*

Securing monitoring and dialogue channels directly within the health center narrowed the locus of response and resulted in quicker changes without waiting for the involvement of the formalized dialogues. At this level, the health center remedied complaints concerning disrespectful treatment of patients during ANC and delivery services by planning respectful maternity care (RMC) and human rights training for health care workers within Continuing Medical Education (CME) sessions. Health care workers were receptive to these trainings and acknowledged that previous CME sessions did not focus on RMC within a human rights framework. Leadership at the refugee health center also revised policies and practices resulting from direct engagement with the Council adolescent representative. As explained by the facility in-charge: *“we had more engagement with adolescents during the implementation of this project which enabled us to lead more adolescent friendly services*, *which we did not before*.*”* Open infrastructure that remains from the emergency phase of the crisis could not be reconditioned during piloting to be more private due to lack of resources. However, the health center worked in collaboration with the adolescent representative to revise service scheduling so that blocks of time on two days per week were reserved for adolescent services and counselling. This improvement was markedly important for pregnant adolescents that described experiencing stigma from other patients when presenting to the facility for ANC. The facility in-charge also reformed operating policies to improve procedures within the medical supplies dispensing unit, to reduce patient wait times. Relatedly, this was notable for adolescents who reported to leave the facility if wait times were long, since confidentiality during the consultation could not be maintained afterwards in public seating areas. Girls and adolescents also received menstrual hygiene kits following revision of distribution patterns by a humanitarian services actor after the actor received complaints that their dissemination did not reach adolescents residing in the margins of the refugee settlement.

The most common challenge duty-bearers and health system actors cited for being unable to bring about an effective remedy was resource constraints; and many expressed frustrations with identifying gaps but limitations in *“taking action because of the resources available*.*”* Although not all complaints were remedied during the pilot period, these broader findings suggest community-led accountability processes can hold health systems accountable and facilitate change in SRHR-related policy and practice.

### Acceptability of rights-based social accountability models within humanitarian coordination structures and among implementing partners

The interrelating coordination of operations in the refugee settlements meant that several actors were engaged in the intervention. At the intervention outset there was initial resistance to establishing a social accountability mechanism that would potentially affect all health and humanitarian system actors providing services and information in the settlements. However, over the course of the intervention there was a clear shift from resistance to acceptance, most notably among health service providers who noted how the approach to addressing and remedying complaints focused on changing systems rather than blaming individual health workers; resourced capacity building for frontline workers; and elevated issues to duty-bearers beyond the health center level through an independent ombudsperson A key informant leading a large-scale family planning program in the settlements reflected on this change as part of her experience participating in the intervention from inception to closure:

*“I think the first time we had resistance was when we sat at their roundtable [during the design phase] when we came on board… I think they [health system actors] looked at it as being a spy on them*. *But after engaging and participating in these engagements and feedback sessions they have come to appreciate the project*. *And they were even making suggestions of scaling to all the health centers so that we can be held accountable for all our service provision*.*”*

Two consistent categories emerged to describe acceptability among humanitarian actors. First, humanitarian partners expressed a shared commitment to norms and principles, which encouraged their participation. According to a national NGO implementer, the proximity of partners working together through SRHR Working Groups and Health and Protection sector coordination meetings generated a shared sense of “*collective rights and responsibilities”* to improve access to quality SRH services when complaints emerged, *“I think it is not surprising*. *To me it is expected that stakeholders and partners working in a setting like this would want to see the feedback from the community on how various services are going*.*”*

Second, humanitarian partners overwhelmingly concluded that the accountability mechanism’s documentation and monitoring bolstered their organizational performance measurement. There was a broad uptake of the community complaints as data to inform the planning and adaptation of health services. Program managers at district-level facilities particularly valued community monitoring data to know *“how our services affect their [refugees] right to access services and as a health system identify gaps in our planning of service provision for refugees especially*.*”* A clinical lead from a leading primary health center described how the intentional inclusion of adolescents within the accountability mechanism enabled the facility to reach contraceptive uptake targets:

*“In the beginning we could not even measure the uptake of contraceptives among adolescents because it was almost negligible*. *It was very low*, *but when this rolled out and we engaged with adolescents and with this feedback we were able to see great change and the proportion of the adolescents’ accessing contraceptives*. *I think in the most recent quarter it increased up to about 2*.*6%*, *which is so great for us as a program*. *It looks small but it is very significant compared to the past when we could not even measure because the uptake was so low*.*”*

A senior health systems manager from the same organization confirmed that their organization’s engagement and learnings from the intervention led to institutionalizing components of the accountability mechanism within their facilities and routine health service delivery model:

*“We used this as an experience to scale up (this approach) in our different operations*. *We have done this in two facilities so far from the time we learned the relevance of this project*. *In the two facilities we needed this programming there was a positive response from the health workers and the commitment to take it up*. *We also got a focal person to oversee at the facility level that the patients’ rights are being respected during service delivery*, *and to remind the patients through health education of what their rights are and what to expect when they come to access services in the facility… we realized if we did not act fast*, *there may be different settlements that could be experiencing this [rights not being respected]*, *but they are not reporting because they don’t have the right mechanism or someone to report to*. *So it really triggered us to move fast*.*”*

As these quotes illustrate, humanitarian implementers were able and willing to engage in social accountability processes to bring about changes in the humanitarian health system. That humanitarian implementers and health systems actors went ahead and integrated components of the community feedback mechanism outside the scope of the pilot and maintained a focus on human rights-based approaches suggests that institutionalizing rights-based accountability is feasible and acceptable in this context.

### Structural barriers and intersecting forms of discrimination affect access to SRH services and holding health systems accountable

Members of the SRHR Council and ombudsperson consistently reflected on the structural barriers and discrimination against women and girls that affected their role as community representatives and monitors. There was a broad understanding that harmful gender norms and stereotypes limited the meaningful participation of women and girls in the accountability mechanism and their access to SRH services more broadly. As illustrated by one representative *“because of what we see in our society… in terms of culture and cultural practice there cannot be change if we do not deal with the root cause and the main dominant actors*.*”* Most frequently cited by participating rights-holders and duty-bearers was how cultural attitudes and gender stereotypes affect lack of uptake for family planning services; and especially how accessing services and participating in open dialogue about SRHR is affected in an environment where lack of male support directly impedes access. The findings also illustrate the compounding effect of conflict on access to SRH services, as described by a refugee women’s representative on the Council:

*“There are some SRH services which the community does not accept and they do not want to hear about it*. *Most especially the family planning services*. *When you come to talk to our people about family planning they will tell you our people have perished too much during the war*, *so there is no need to stop our women from reproducing*. *They have to reproduce to fill the gaps of those ones that died during the war*.*”*

The gendered impact of the COVID-19 pandemic is evident in the complaints collected by the intervention structures and corroborated by independent reporting on increasing early marriage and unintended adolescent pregnancy in the district. Access to acceptable and quality adolescent contraceptive counselling, ANC, and delivery services with an aim to prevent unintended pregnancy were remedied during the intervention. However, the Council in consultation with the ombudsperson also recommended measures to go beyond the locus of SRH service delivery and to reform sub-county level by-laws to address early marriage and harmful gender norms in line with national-level legislation and international and regional human rights obligations. By-laws are the highest level of laws at the sub county level and are debated by local councils elected to lead the sub-county; they are perceived as closer to the community and their implementation as more accessible to women and girls in the settlements. This response requires a longer-term advocacy and legislative process and was not actioned during piloting. However, mobilization of the community structures to demand accountability from duty-bearers through legislative measures signals the social accountability mechanisms’ potential for engaging in reform at that level.

## Discussion

Our findings demonstrate how a human rights-based approach, which prioritizes a broad and robust understanding of accountability, can be operationalized to hold humanitarian and host health systems to account in a specific refugee and post-conflict context. While the intervention focused on social accountability and did not seek to bring about remedies through traditional legal or human rights mechanisms, examination of the findings demonstrates how human rights strategies, standards, and principles can generate clarity on which to measure accountability for health and human rights. Developing accountability strategies for SRHR that recognize the complementary and mutually reinforcing law and policy across the human rights, development, and humanitarian nexus is a promising approach to strengthen accountability for the increasing number of women and girls affected by contemporary forms of crisis, forced displacement, and development-humanitarian contexts such as northern Uganda [17]. As a starting point, grounding the community mechanism in human rights principles limited the extent to which deliberative decision-making processes based on prevailing power dynamics shaped SRHR priorities, as is well documented in the social accountability and human rights literature [43]. Indeed, the mere application of standards alone, without deep contextualization of setting and situation, risks reducing such approaches to a checklist exercise and is insufficient to bring about sustainable and legitimate remedies, especially in contexts where traditional access to justice and legal accountability are challenged. However, sensitizing human rights standards and principles through community awareness sessions and peer solidarity groups in the refugee and host community supported the development of an awareness of SRHR and other entitlements, including through the intervention structures. Scoping research on accountability strategies in humanitarian settings suggests that there is often less focus on programs and policies to “develop a critical consciousness” within affected populations about their rights [15]. Our findings demonstrate the importance of this approach as part of realizing accountability for SRHR in this context as both a process and an outcome.

The open-ended and participatory approach to the community complaints mechanism resulted in refugee and host women and girls attributing meaning to the health and rights issues that were important to them. Maintaining an indeterminate focus and not being prescriptive about what the intervention structure would capture also allowed the mechanism to respond to emergent challenges and issues. This was especially salient throughout the era of COVID-19 in Uganda, where characteristics of complaints fluctuated along with the closure of schools and movement restrictions affecting access to essential medicines, which emerging evidence on the impact of the pandemic on SRHR validates [8, 44]. Complaints on issues affecting access to SRH services ranged from nutrition to intimate partner violence (IPV) and further demonstrates the multiple and intersecting factors that impact the claiming of SRHR in humanitarian situations [45]. Moreover, that GBV was one of the most frequently reported complaints to the community mechanism may suggest that it was more acceptable to women and girls to confide in the women, adolescent, and disability representatives rather than other formal protection mechanisms. This finding is perhaps unsurprising since it is well documented that persons are more likely to report GBV to a trusted party and that there are specific challenges associated with GBV reporting and access to comprehensive clinical care and justice [46]. Nonetheless, it corroborates evidence of the broader importance of having trusted intermediaries in the community to monitor and demand accountability for health-related human rights violations [47]. The intervention did not receive complaints related to lack of access to safe abortion within the district health system, despite concerns identified throughout the study over high rates of unsafe abortion in northern Uganda and sensitization on access to safe abortion in line with regional and international legal obligations of the government of Uganda [31, 48]. Significant barriers affect access to abortion services and information in humanitarian settings and the findings highlight the need for efforts to inform, increase access, and destigmatize safe abortion among both rights-holders and duty-bearers in future interventions focused on comprehensive SRHR.

### Developing strategies

Part of the hypothesis of the intervention is that a human rights-based approach ensures that the capacity of both duty-bearers and rights-holders is built from the onset to understand their obligations and existing entitlements, respectively. Anticipating areas where misconceptions or negative perceptions about human rights might emerge among duty-bearers and/or health systems actors was an effective strategy as part of design and implementation. Specifically, the ombudsperson’s routine liaison and monthly dialogues with public authorities and humanitarian agencies facilitated a process of identifying and addressing knowledge and capacity gaps through constructive dialogue about the content of human rights standards and principles that underpin human rights accountability. Debate about the value of establishing formal institutional-level roles for a humanitarian ombudsperson continues [49]. However, the concept of the intervention-level ombudsperson was focused on testing whether independent third parties selected through social processes of dialogue and negotiation with duty-bearers and rights-holders could build trust to adjust power dynamics and bring about remedial action when SRHR is not respected. The acceptability and willingness among most duty-bearers to engage in the intervention aligns with observations from other social accountability studies, including some studies that demonstrate how the sharing of norms and behaviors associated with organizational commitments can enhance interpersonal accountabilities among humanitarian actors [50].

While more evidence is needed on effective ways to institutionalize rights-based accountability for SRHR across integrated humanitarian health systems in northern Uganda, incorporation of Council representatives and the ombudsperson within existing structures was important for sustaining intervention changes and ensuring the continued participation of traditionally excluded groups in decision-making processes. Refugee Welfare Committees and Councils (RWC) and HMUs are well-recognized decision-making structures in other protracted humanitarian contexts. Yet, as is the case in Adjumani, these systems often lack an adequate sub-group or focus on comprehensive SRHR. Bringing stigmatized SRHR issues and concerns of the most marginalized groups into the public domain using these fora and trusted intermediaries may be valuable for institutionalizing norms and practice changes over time and would respond to recognized limitations of social accountability processes [51].

Broader strategies for success that this pilot shares with other interventions focusing on accountability for comprehensive SRHR among marginalized populations are explored elsewhere; and suggest that linking initiatives to institutionalized processes, such as legal accountability, and intentionally including marginalized populations through distinct roles and processes are key strategies that may be applied more broadly and to scale.

### Challenges

Programmatic reflections on the overarching success and challenges of this intervention are documented elsewhere [43]. However, the most persistent challenge specific to the context was sustaining social change within the transitional nature of humanitarian response that is shaped by high turnover, aid dynamics, and frequent transferring of humanitarian program staff [16, 49]. This phenomenon was equally relevant in the host community, as the intervention was implemented during a national general election. Several duty-bearers that had championed the rights-based social accountability mechanism and participated in the ombudsperson selection process were not re-elected. In some cases, change in leadership met the pilot with resistance requiring re-establishing an understanding and legitimacy of the structures. Building the capacity and will of duty-bearers beyond identified responsible actors at the time, is an important consideration for future interventions.

## Limitations

This study has limitations associated with pilot studies in general, including the limited scale of the study site and short period (15 months) of implementation and observation. Longitudinal qualitative methods were used to capture the in-depth and changing perspectives and behaviors of participants. However, these methods do not intend to yield representative or generalizable results of all refugee contexts in Uganda, or other humanitarian settings. Through designing the study with and interviewing stakeholders across the humanitarian and human rights sector and geographies, we posit certain core elements of the findings are transferable beyond the study setting and population so long as they are context specific.

Participant responses elicited throughout the study trended towards positive and may introduce a social desirability bias. The potential for participants to overstate change and commitments to remedy and response was anticipated by the study team, based on findings from other social accountability interventions [36, 52]. This was minimized during data collection by working with local research assistants not affiliated with the lead implementing organization to conduct in-depth interviews. Furthermore, interviews with government and humanitarian duty-bearers were conducted by members of the study team who were not involved in field operations or implementation. To increase credibility and trustworthiness, the multi-method design allowed for triangulation of data; and findings were further validated against data sources provided by health implementing partners operating in the context and official government and UN agency sources, where possible.

The extent to which there were irregularities or inaccurate reports in the complaint data at community-level is unknown. To avoid duplication of complaints from the same person, program structures were trained to document this in the complaint logbook if this issue arose. While the study team reviewed and validated the tracker on a bi-weekly basis, some complaints were collected from an aggregation of women and girls participating in peer solidarity groups due to confidentiality concerns. Therefore, it is possible that multiple observations from an individual are not fully excluded. Follow up research with participants from the pilot study would support an understanding of the sustainability and embeddedness of changes beyond the pilot phase and any longer-term benefits of having participated in the rights-based social accountability structures. Finally, piloting focused on establishing the feasibility of implementation and acceptance within the context; and was not designed to measure changes in SRH services use, uptake, behavior change, or health outcomes. Despite a range of rights-holders and duty-bearers attributing health-related changes, such as decreased incidence of unintended adolescent pregnancy, these claims cannot be confirmed using the existing data.

Remedies effected by local government and humanitarian response structures during the intervention, including the RWC and HMUs, reflect the Pagirinya settlement and Adjumani district level politics that influence community dynamics and SRHR. Building the political will and capacity of health duty-bearers to understand and actively engage with rights-based accountability was a central component of the project, however different intervention logic may be required for achieving similar results and meaningful adaptation in other implementation contexts, which is consistent with a human rights-based approach to program design. Initiatives to implement and adapt learnings from this human rights project in other humanitarian settings are ongoing and in future will support a better understanding of which strategies and components of this context-specific project can be generalized and applied across other global settings.

## Conclusion

This pilot study contributes to the growing body of literature on accountability strategies for SRHR in humanitarian settings and the importance of centering a human rights-based approach in their implementation. Strengthening integrated humanitarian health systems to be more accountable to the needs and human rights of affected women and girls is critical for delivering transformative change and realizing SRHR in these complex contexts. Results from the community-led intervention demonstrate the feasibility and acceptability of embedding these designs in the refugee response infrastructure; promising approaches to ensure the effective participation of all women and girls; strategies to mitigate risk through independent intermediaries determined by affected communities; and building the capacity of both rights-holders and duty-bearers to exercise their rights and meet their obligations. Implementing this intervention in the context of northern Uganda also offers distinctive lessons for applying rights-based social accountability strategies across the humanitarian, development, and human rights nexus [17, 53]. As more people are displaced and humanitarian crises become increasingly protracted, innovative strategies for the realization of SRHR are needed and should continue to be examined in future research.

## Supporting information

S1 Fig Map of refugee settlements and total refugees and asylum-seekers in Uganda, UNHCR refugee population statistics database, 2022. (PDF)

S1 Table Intervention components and description. (DOCX)

S1 Text Interview guides. (DOCX)
