# Supplementary figures and images for: Human rights-based accountability for sexual and reproductive health and rights in humanitarian settings: Findings from a pilot study in northern Uganda
Source: PLOS Glob Public Health. 2022 Aug 22;2(8):e0000836. doi: 10.1371/journal.pgph.0000836 (PMC10021271; doi:10.1371/journal.pgph.0000836)

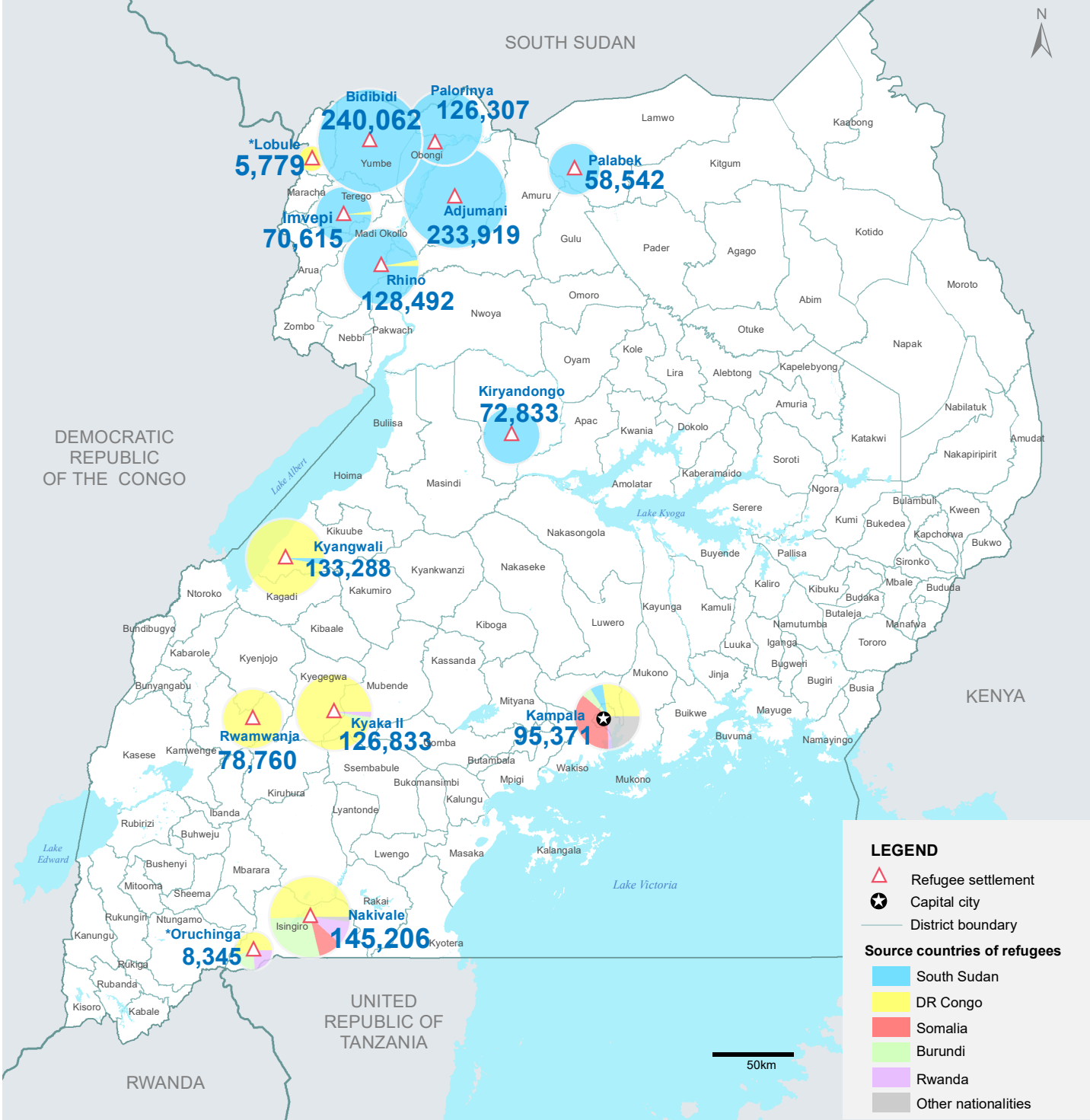

Supplement: S1 Fig — (PDF) [file pgph.0000836.s001.pdf]
