# Supplementary material for: Human rights-based accountability for sexual and reproductive health and rights in humanitarian settings: Findings from a pilot study in northern Uganda
Source: PLOS Glob Public Health. 2022 Aug 22;2(8):e0000836. doi: 10.1371/journal.pgph.0000836 (PMC10021271; doi:10.1371/journal.pgph.0000836)
Supplement: S1 Table — (DOCX) [file pgph.0000836.s002.docx]

**Intervention components: participant characteristics, recruitment, and mandate**

| **Intervention structure** | **Participant characteristics** | | **Sampling and recruitment strategy** | **Role and mandate** | **Associated participation in research activities** |
| --- | --- | --- | --- | --- | --- |
| **Council for SRHR** | ***N*** | 9 | Representatives were purposively selected based on in-depth interview data collected during the design phase and snowball sampling. Representatives were identified and directly approached through their existing leadership positions on Refugee Welfare Committees, Local Councils, and women, adolescent, and disability community peer-groups. One representative was a health care worker from a refugee settlement health center and nominated by health center management to participate in the accountability mechanism on behalf of health system actors. | Representatives were trained to sensitize and build a shared understanding of human rights and SRHR within the community and document complaints in the complaint logbook. Representatives were mandated to confidentially collect and review complaints received from women and girls in the settlements and refer complaints to the ombudsperson, or other district or camp level mechanisms for non-SRHR complaints. | Accountability ecosystem mapping  Complaint logbook entry and document review  In-depth interviews  Focus group discussion |
|  | **Sex** |  |  |  |  |
|  | Female | 8 |  |  |  |
|  | Male | 1 |  |  |  |
|  | **Age (years)** |  |  |  |  |
|  | < 18 | 3 |  |  |  |
|  | 19-25 | 3 |  |  |  |
|  | 26-49 | 3 |  |  |  |
|  | **Migration status** |  |  |  |  |
|  | Refugee | 5 |  |  |  |
|  | Host | 4 |  |  |  |
| **Ombudsperson** | ***N*** | 1 | District-level government duty bearers and humanitarian health system actors led a recruitment process to select an independent third party from within the refugee or host community. Following an open call for the position, candidates participated in a written exercise and interview over a one-day period. Duty-bearers used consensus-based decision-making to select the successful candidate. | Role is to serve as an independent intermediary between rights-holders (refugee and host community) and duty-bearers (district government and humanitarian actors). The ombudsperson was conferred a mandate to support the Council for SRHR collect complaints, review complaints, give explanations for decisions taken, and support duty-bearers to facilitate access to effective remedy when rights are violated. | Accountability ecosystem mapping  Complaint logbook entry and document review  In-depth interviews |
|  | **Sex** |  |  |  |  |
|  | Female | 1 |  |  |  |
|  | Male | 0 |  |  |  |
|  | **Age (years)** |  |  |  |  |
|  | < 18 | 0 |  |  |  |
|  | 19-25 | 0 |  |  |  |
|  | 26-49 | 1 |  |  |  |
|  | **Migration status** |  |  |  |  |
|  | Refugee | 0 |  |  |  |
|  | Host | 1 |  |  |  |
| **Community monitors** | ***N*** | 7 | Community monitors were purposively sampled from the refugee and host community based on their pre-existing participation as a health volunteer with ongoing health programs operating in Adjumani district and refugee settlements. | Mandated to monitor implementation of response at community-level after complaints are reviewed and decisions for action are taken by duty-bearers. Community monitors also sensitize information about accountability and SRHR during community-level activities, including disseminating media and information, engaging youth and community groups in workshops, door-to-door sensitization, and referring complaints and feedback to the Council. | Complaint logbook entry and document review  Focus group discussion |
|  | **Sex** | 7 |  |  |  |
|  | Female | 7 |  |  |  |
|  | Male | 0 |  |  |  |
|  | **Age (years)** |  |  |  |  |
|  | < 18 | 0 |  |  |  |
|  | 19-25 | 0 |  |  |  |
|  | 26-49 | 7 |  |  |  |
|  | **Migration status** |  |  |  |  |
|  | Refugee | 5 |  |  |  |
|  | Host | 2 |  |  |  |
