# Supplementary material for: Human rights-based accountability for sexual and reproductive health and rights in humanitarian settings: Findings from a pilot study in northern Uganda
Source: PLOS Glob Public Health. 2022 Aug 22;2(8):e0000836. doi: 10.1371/journal.pgph.0000836 (PMC10021271; doi:10.1371/journal.pgph.0000836)
Supplement: S1 Text — (DOCX) [file pgph.0000836.s003.docx]

**Interview Guide A: Key informants (duty-bearers)**

**Introduction**

- Introductions and thank you for your time.
- Explain objectives and expectations of interview.
- Obtain informed consent to record the interview (read ethics policy).
- Explain the future purposes, use, attribution, and data storage information.

**Accountability Mechanism: Participation and outcomes**

1. Thank you for your engagement in the SRHR Accountability Pilot. In the last year, what have been the most significant changes resulting from the project?
   1. *P1: quality of SRHR services?*
   2. *P2: Pagirinya settlement community?*
   3. *P3: your role as an SRHR duty-bearer?*
2. What SRHR issues has this project addressed well?
   1. *P1: General SRHR and subtopics*
   2. *P2: Influencing factors*
3. What SRHR issues does this project not addressed well?
   1. *P1: General SRHR and subtopics*
   2. *P2: Influencing factors*
4. How has the project enabled your role as a duty-bearer to respond to SRHR complaints?
   1. *P1: SRHR obligations, capacity building, hold to account*
   2. *P2: why/why not*
5. In your role as an SRHR duty-bearer, what has been challenging, or created barriers for your engagement throughout the project?
   1. *P1: influencing factors*
   2. *P2: why/why not?*

**Expanding and institutionalizing**

1. Is there a need and/or interest in expanding this project in other settlements across Adjumani?
   1. *P1: why/why not*
   2. *P2: other SRHR stakeholders or coordination partners*
   3. *P3: availing resources*
2. How can the SRHR accountability structures be integrated into existing accountability mechanisms, or interventions in Pagirinya/Adjumani?
   1. *P1: structures: health centers admin, HMU, RWC 1-5, SRMH Technical Working Groups*
   2. *P2: why/why not*

**Open Feedback and conclusion**

Thank you very much for your participation as a key informant. Those are all my interview questions.

1. Do you have any other feedback, or recommendations for action that you have not yet shared that you would like to add?
2. Is there anyone else you recommend we speak to during this research?
3. Do you have any other questions for me?

**Interview Guide B: Key informants (rights-holders/accountability mechanism representatives)**

**Introduction**

- Introductions and thank you for your time.
- Explain objectives and expectations of interview.
- Obtain informed consent to record the interview (read ethics policy).
- Explain the future purposes, use, attribution, and data storage information.

**Implementation: Effectiveness and change**

1. In the last year of programming, what do you think have been the most significant changes resulting from the project?
   1. *P1: In the quality of SRHR services?*
   2. *P2: In the quality of women and girls’ sexual and reproductive lives?*
   3. *P3: In Pagirinya settlement community?*
   4. *P4: In your participation as a (Council member or Community Based Facilitator)?*
2. Do you think the actual project outcomes align with the expected outcomes (see TOC and logic model)?
   1. *P1: Why/why not?*
   2. *P2: Influencing factors*
3. To what extent do you think the pilot delivered against the SRHR needs and human rights of women and girls in Pagirinya settlement?
   1. *P1: Scale, size, approach, rationale, impact, efficiency, integration*
4. In your opinion, what factors ensured full and effective implementation of the pilot?
   1. *P1: Why/why not?*
   2. *P2: Expected/unexpected influencing factors*
5. What factors presented challenges (barriers) to enabling implementation?
   1. P1: *Why/why not?*
   2. *P2: Expected/unexpected influencing factors*
6. Describe (if any) the factors that differentiated this pilot from other humanitarian programs?
   1. *P1: Why/Why not*
   2. *P2: influencing factors within and outside of existing humanitarian service provision.*

**Recommendations: Expansion and institutionalizing**

1. In your opinion, what changes would improve future scaling or replication of this project design?
   1. *P1: design, implementation, resources, research, stakeholders, target population*
2. How can the SRHR accountability structures be better integrated into existing accountability mechanisms, or interventions in Pagirinya/Adjumani?
   1. *P1: Structural: existing programming, health centers admin, HMU, RWC 1-5, SRMH TWG*
   2. *P2: Why/why not*

**Open Feedback and conclusion**

Thank you very much for your participation as a key informant. Those are all my interview questions.

1. Do you have any other feedback, or recommendations for action that you have not yet shared that you would like to add?
2. Is there anyone else you recommend we speak to during this research?
3. Do you have any other questions for me?

**Focus Group Discussion Guide: Refugee and host community (rights-holders and accountability mechanism representatives)**

**Introduction: SRH service need and uptake**

1. What would you describe as the primary SRH needs in your community?
   1. *Probes: Major SRH events, issues, concerns, problems.*
2. How do you receive information about SRH services and where do you access services?
   1. *P1: Clinics, traditional attendant, district facilities.*
   2. *P2: Experiences, positive, negative.*
   3. *P3: Why do you seek services at these places?*
   4. *P4: referral services; to receive elsewhere*

**Human Rights Framework: AAAQ**

1. Do you think that the services you’ve just described are:
   1. *Available – examples.*
   2. *Accessible – examples.*
   3. *Acceptable – examples.*
   4. *Good quality – examples.*
      1. *P1: goods, services, facilities, resources, programs*
      2. *P2: barriers/stigma*

**Human Rights Framework: Participation and inclusion**

1. How are you included and able to participate in the decisions that are made about SRH services provided?
   1. *P1: opportunities to participate fully, meaningfully, and effectively.*

**Human Rights Framework: Accountability**

1. Are there systems in place for you to provide feedback or make a complaint about the SRH services you receive?
   1. *P1: have you used these mechanisms; what was the outcome?*
   2. *P2: effectiveness, timely, remedy, change in program or policy.*
   3. *P3: how did you learn about these systems?*
2. Tell me what you think could be done to improve these systems?
   1. *P1: design needed for empowered access.*
   2. *P2: comfortable reporting to.*
   3. *P3: referral process.*

**Conclusion**

We are all done. Thank you all very much for taking the time to speak with us today.

1. Do you have any other feedback, or recommendations that you have not yet shared that you would like to add?
2. Do you have any other questions for me?
3. If anyone would like to speak one on one and confidentiality about the topics we covered today, please feel free to come see (*name of facilitator and translator*) at the end of the discussion.
